# Supplementary material for: Automatic Evaluation for Bioengineering of Human Artificial Ovary: A Model for Fertility Preservation for Prepubertal Female Patients with a Malignant Tumor
Source: Int J Mol Sci. 2022 Oct 17;23(20):12419. doi: 10.3390/ijms232012419 (PMC9604043; doi:10.3390/ijms232012419)
Supplement: Supplementary file 1 [file ijms-23-12419-s001.zip › Figure S1.pdf]

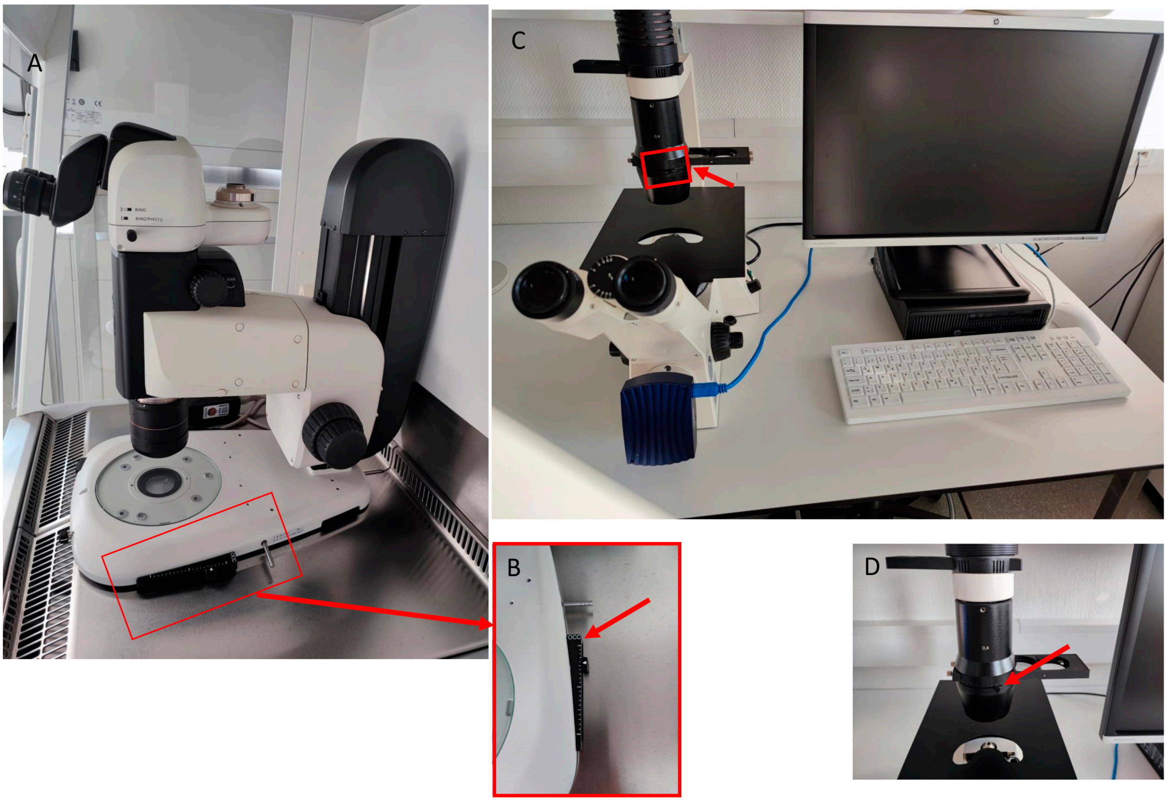

Figure S1. Optical microscope setup parameters. (**A**). Nikon SMZ18 Stereoscope setup in oblique coherent contrast (OCC) mode; (**B**). detailed setup of stereoscope under OCC illumination: the arrow shows the exact scales on the slide lever for perfect bright field imaging; (**C**). Zeiss Axiovert 40CFL microscope setup using phase contrast (Ph) mode; (**D**). detailed position of the aperture under Ph0.4 mode. The supplementary video is an example of a 3D view of the artificial ovary stained with RedDot.
